# S-nitrosylation contributes to ER stress and aggresome formation in secosterol-B-mediated endothelial dysfunction

**DOI:** 10.1016/j.jlr.2026.101017

**Published:** 2026-03-10

**Authors:** Maria Gemma Nasoni, Erik Bargagni, Francesca Monittola, Pasquale Creanza, Elisa Maricchiolo, Sofia Masini, Anastasia Ricci, Serena Benedetti, Silvia Carloni, Sabrina Burattini, Alessandra Fraternale, Andrea Pompa, Michele Menotta, Luigi Iuliano, Rita Crinelli, Francesca Luchetti

**Affiliations:** 1 Department of Biomolecular Sciences, University of Urbino Carlo Bo, Urbino, Italy; 2 Department of Medico-Surgical Sciences and Biotechnology, Sapienza University of Rome, Rome, Italy

**Keywords:** HUVECs, oxysterols, atherosclerosis, nitric oxide, nitrosative stress, UPR, GRP78, misfolded proteins, proteomics

## Abstract

The involvement and the *in vivo* relevance of endoplasmic reticulum (ER) stress in the atherosclerotic process are well established, but the mechanisms have been only partly elucidated. Emerging evidence indicates that ER protein folding pathways are sensitive to nitric oxide (NO) fluctuations and therefore heavily vulnerable under conditions of nitrosative stress. Recent research indicates that protein S-nitrosylation (S-NO), a key redox-mediated modification involved in several disorders, affects neuronal function by altering ER stress sensor proteins. However, the mechanisms by which ER protein S-NO impacts vascular diseases remain unclear. Here, we provide evidence that secosterol-B (SEC-B), an oxysterol found in atherosclerotic plaques, induces nitrosative stress and protein S-NO in vascular endothelium, leading to ER stress. In detail, our findings demonstrate that SEC-B triggers activation of the inositol-requiring enzyme-X-box binding protein 1 signaling pathway and causes ER-membrane expansion and the accumulation of misfolded proteins in human umbilical vein endothelial cells. In parallel, increased NO levels with upregulation of inducible nitric oxide synthase protein expression and alterations in the nitrosylation levels of various proteins, including protein disulfide isomerase and glucose regulatory protein 78, were observed. Interestingly, pretreatment with NG-nitro-L-arginine methyl ester strongly reduced ER swelling and aggresome formation. Collectively, our findings demonstrate that NO and protein S-NO play a critical role in SEC-B-induced ER dysfunction, providing new insights into the mechanisms underlying vascular dysfunction observed in atherosclerosis and highlighting potential therapeutic targets to preserve endothelial integrity.

## Graphical abstract

**Figure:**
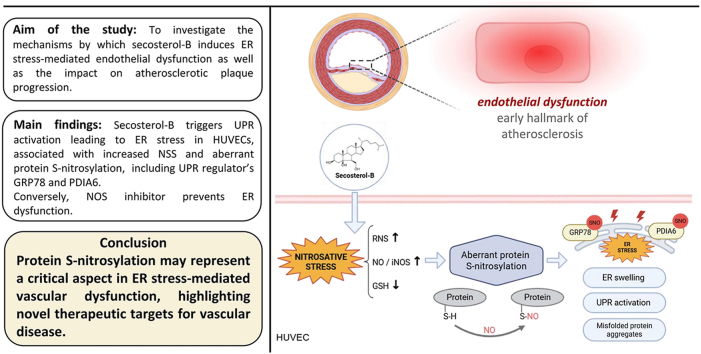

Endothelial cells play a central role in regulating key physiological processes, including vascular tone, shear stress response, and inflammation. Dysfunction of the endothelium is recognized as an early event in the development of atherosclerosis and coronary artery disease, increasing the risk of cardiovascular outcomes.

It is well established that oxidized LDLs (oxLDLs) play a critical role in both the initiation and progression of atherosclerosis. During oxidative modification of LDL, various products are generated, including oxysterols, lysophospholipids, and isoprostanes. In particular, oxysterols are a major component of oxLDL generated through the enzymatic and nonenzymatic oxidation of cholesterol (1, 2).

At physiological concentrations, oxysterols contribute to the regulation of diverse biological processes by modulating cell signaling and gene expression. On the other hand, an abnormal accumulation of oxysterols has been associated with chronic diseases, such as neurodegenerative disorders, cardiovascular diseases, autoimmune conditions, and various metabolic disorders (3). Notably, elevated oxysterol levels have been detected in the blood of hypercholesterolemic individuals and in atherosclerotic plaques (4, 5).

Like other lipid oxidation products, oxysterols can contribute to cell dysfunction due to their proinflammatory, pro-oxidant, proapoptotic, and fibrogenic properties (2). Secosterol-B (SEC-B) is one of the major ozonolysis products of cholesterol (1); it is present at high levels in human LDL, atherosclerotic plaques, and brain tissues affected by neurodegenerative diseases (6, 7).

Our previous work showed that SEC-B impacts endoplasmic reticulum (ER) in human umbilical vein endothelial cells (HUVECs), exerting proapoptotic effects at high concentrations while inducing protective autophagy at lower concentrations to promote cell survival (8). However, the precise mechanisms by which SEC-B contributes to endothelial dysfunction and its role in the pathogenesis of atherosclerosis remain largely unclear.

Recent studies have emphasized the importance of ER stress in both the initiation and progression of atherosclerosis (9, 10). Disturbances in ER homeostasis lead to changes in the ER redox state and/or the rate of protein synthesis exceeding the rate of folding, causing an accumulation of unfolded proteins within the ER, resulting in a condition of ER stress. This, in turn, triggers the unfolded protein response (UPR) initiated by the activation of three ER transmembrane proteins, that is, inositol-requiring enzyme 1 (IRE1), protein kinase RNA-like endoplasmic reticulum kinase (PERK), and activating transcription factor 6 (ATF6) (11). The primary role of UPR is to protect the cell from ER stress by inducing the transcription of genes encoding ER chaperones, that is, the glucose regulatory protein 78 (GRP78) and enzymes that promote protein folding, maturation, trafficking, and ER-associated protein degradation so as to remove the accumulated misfolded proteins in the ER. Quality control in the ER protects cells from the accumulation of aberrant proteins, and their subsequent autophagic clearance represents a compensatory protein degradation mechanism for proteasomal failure (12).

However, if the cell fails to clear up the protein-folding defect and restore ER homeostasis, the UPR will trigger apoptosis to eliminate the stressed cells. Several pieces of evidence highlighted that chronic activation of the UPR has been implicated in a variety of human cardiovascular pathologies through the induction of endothelial dysfunction. During atherosclerosis, oxLDL induces the UPR and oxidative stress in the endothelium by inhibiting sarcoplasmic/ER Ca2+-ATPase (13). Among these processes, oxLDL induces apoptosis, primarily via the PERK/eukaryotic initiation factor 2 alpha/CCAAT/enhancer-binding protein homologous protein pathway (14).

Moreover, emerging evidence suggests that protein-folding pathways within the ER are sensitive to changes in nitric oxide (NO) levels (15). It has been demonstrated that permanently elevated levels of NO can be linked to inflammatory conditions associated with pathological disorders (15). Nevertheless, the mechanisms by which NO activates ER stress pathways in the context of atherosclerosis have not been fully elucidated.

Excessive production of reactive nitrogen species (RNS), including NO, combined with GSH depletion, leads to a condition known as nitrosative stress (NSS), which contributes to cellular damage or death. NSS has been implicated in several cardiovascular pathologies, including myocardial ischemia/reperfusion injury, aortic aneurysm, heart failure, hypertension, and atherosclerosis (16).

Protein S-nitrosylation (S-NO) is a redox-mediated modification that occurs under NSS conditions. This modification leads to the covalent binding of NO to a cysteine thiol, forming S-nitrosothiols, regulating protein function, stability, localization, and interactions, and playing a key role in cellular signaling (17).

Aberrant S-NO can have detrimental effects on the cells and has been linked to a variety of pathophysiological processes, including cardiovascular disease. Notably, under NSS conditions, ER stress sensors, such as IRE1α, PERK, and/or ER-resident protein isomerases, have been found aberrantly S-nitrosylated in models of neurodegeneration (18). However, the role of protein S-NO in ER stress-mediated atherosclerosis development remains largely unexplored.

In this study, we investigate the mechanisms by which SEC-B modulates the UPR in HUVECs, focusing on the role of protein S-NO in the initiation of ER stress. Our findings aim to provide new insights into the pathophysiological processes underlying atherosclerosis and identify potential targets for prevention and therapy.

## Materials and methods

### Cell culture and treatment

HUVECs (Merck-Sigma-Aldrich, catalog no.: SCCE001) were cultured in EndoGRO™ Basal Medium with the addition of supplemental factors (EndoGRO-LS, Merck-Sigma-Aldrich, catalog no.: SCME001) and 1% streptavidin-penicillin in a humidified atmosphere of 5% CO_2_ at 37°C. 3β-hydroxy-5β-hydroxy-B-norcholestane-6β-carboxaldehyde (SEC-B) was synthesized by cholesterol ozonation (19).

At 80% confluence, cells were detached with trypsin-EDTA, washed, and subcultivated in new flasks for 1-2 days before the experiments. HUVECs were treated with 10 μM SEC-B, and at the end of the incubation time, cells were washed with PBS and stained with fluorophores or submitted to cell lysis as detailed below. In experiments involving inhibition, cells were pretreated with 100 μM NG-nitro-L-arginine methyl ester (L-NAME; EMD Millipore Corp, catalog no.: 483125) for 1 h prior to SEC-B treatment.

### Human specimens

Human carotid samples were obtained from patients undergoing carotid endarterectomy because of carotid atherosclerosis with lumen stenosis >70%. Patients gave written informed consent, and all procedures involving sampling were approved by the Institutional Ethics Committee. The study was conducted in accordance with the ethical principles outlined in the Declaration of Helsinki.

Tissue samples were rinsed with cold S-NO Wash Buffer (1X) for 10 min and fixed with 4% paraformaldehyde at 4°C overnight under gentle agitation.

Samples were washed three times for 5 min with S-NO Wash Buffer (1X), frozen, and cryoembedded in OCT compound before being cut with a cryostat at a thickness of 10 μm and fixed on slides.

### Oil Red O staining

Carotid sections were washed two times with PBS and then with 60% isopropanol for 1 min and left to completely dry in air. Sections were then stained with 0.3% Oil Red O solution in isopropanol at 60% for 1 h, followed by a quick wash with 60% isopropanol and four washes with PBS. Staining was observed with an inverted microscope with data acquisition software (Nikon ECLIPSE TS100 with NIS-Elements F software; Nikon Europe BV, Amsterdam).

### Cell extracts and Western immunoblotting analysis

Cells were processed as previously reported (20). Blots were probed with the following antibodies: anti-spliced X-box binding protein 1 (XBP-1s) (E9V3E, #40435), ATF6 (D4Z8V, #65880), BiP/GRP78 (C50B12, #3177), and protein disulfide isomerase (PDI) (#2446) from Cell Signaling Technology; anti-endothelial NOS (eNOS) (A1548) from ABclonal; and anti-NOS2 (N-20, sc-651) from Santa Cruz Biotechnology. Total protein and immune complex detection were performed in a ChemiDoc MP Imaging System (Bio-Rad). The quantification of the immunoreactive signal was performed using the Image Lab software, version 5.2.1 (Bio-Rad), and the obtained values were normalized on total protein content as determined by the No-Stain reagent.

For redox Western blot analysis, samples were separated by SDS-PAGE under reducing (+β-mercaptoethanol, +MSH) and nonreducing conditions (-MSH), blotted, and stained with an antibody against PDI.

### Immunofluorescence

HUVECs were grown on 35 mm MatTek glass-bottom dishes (MatTek Corporation; density, 2 × 10^5^ cells/well). After treatment, cells were processed for immunofluorescence as previously reported (20). The cells were incubated overnight at 4°C with the anti-GRP78 antibody (C50B12; #3177). After being washed (4X) with PBS, the cultures were incubated for 1 h with a conjugated anti-rabbit secondary antibody. The same protocol was also applied to the carotid sections; in detail, they were incubated overnight with the CD31/PECAM-1 (H3) antibody (sc-376764; Santa Cruz Biotechnology) at 4°C. After being washed (4X) with PBS, the sections were incubated for 60 min with conjugated anti-mouse secondary antibody. Subsequently, cells and sections were washed (4X) with PBS, and fluorescent images were captured using the 63x oil objective by confocal microscopy (Leica TCS SP5 II confocal microscope, Wetzlar, Germany).

### Transmission electron microscopy

For transmission electron microscopy, cells were seeded in 25 cm^2^ flasks. At the end of the treatment, the cells were washed and then fixed for 1 h with glutaraldehyde solution (2.5% in 0.1 M phosphate buffer, pH 7.4). Cells were washed in a phosphate buffer and postfixed in osmium tetroxide (1% in 0.1 M phosphate buffer, pH 7.4) for 2 h. At the end of incubation, cells were repeatedly washed in a phosphate buffer, dehydrated in a graded ethanol series, and embedded in Araldite. Semithin (2 μm thick) and ultrathin sections (70–80 nm thick) were cut with an LKB 2088 Ultratome V. The ultrathin sections were contrasted with UranyLess solution, followed by treatment with lead citrate. The sections were observed under a Philips CM10 transmission electron microscope.

### Quantification of intracellular NSS

NO and RNS production was analyzed in HUVECs by 4,5-diaminofluorescein diacetate probe (5 μM; ThermoFisher) and BioTracker 515 Green ONOO- dye (2.5 μM; SCT035, Sigma-Aldrich), respectively. Briefly, the cells were incubated with fluorescent probes for 30 min at 37°C, and then the fluorescence emission was analyzed at ex/em 485/520 nm in a FluoStar Optima (BMG Labtech, Ortenberg, Germany) multiwell plate reader.

### GSH quantification

HUVECs (1 × 10^6^ cells/flask) were lysed with 100 μl of lysis buffer (0.1% Triton X-100, 0.1 M Na_2_HPO_4_, 5 mM EDTA, pH 7.5), followed by 15 μl of 0.1 N HCl and 140 μl of precipitating solution (100 ml containing 1.67 g [w/v] of glacial metaphosphoric acid, 0.2 g [w/v] of disodium EDTA, and 30 g [w/v] of NaCl). After centrifugation, pellets were resuspended in 100 μl of 0.1 N NaOH, and proteins were quantified via Bradford assay (Bio-Rad). Supernatants were added with 25% (v/v) 0.3 M Na_2_HPO_4_ and 10% (v/v) DTNB for thiol determination by HPLC through a BDS Hypersil™ C18 column (5 μm, 150 × 4.6 mm; Thermo Scientific). Separation and elution conditions were previously described elsewhere (21). Following the detection at 330 nm, quantitative measurements were compared with known concentration standards and normalized to the protein content.

### ER staining

HUVECs were grown on 35 mm MatTek glass-bottom dishes (density, 2 × 10^5^ cells/well). After treatment, HUVECs were washed and incubated in HBSS 100 nM ER-Tracker Green (ThermoFisher) for 30 min at 37°C. Fluorescent images were observed and captured by confocal microscopy using a 63x oil objective (Leica TCS SP5 II confocal microscope, Wetzlar, Germany).

### NO staining

HUVECs were grown on 35 mm MatTek glass-bottom dishes (density, 2 × 10^5^ cells/well). After treatment, HUVECs were washed and incubated in HBSS with 5 μM 4,5-diaminofluorescein diacetate (ThermoFisher) for 30 min at 37°C. Fluorescent images were observed and captured by confocal microscopy using a 63x oil objective (Leica TCS SP5 II confocal microscope, Wetzlar, Germany).

### Detection of aggregated proteins

Aggregated proteins were detected using the Proteostat Aggresome Detection Kit (Enzo, catalog no.: ENZ-51035) according to the manufacturer's instructions. Briefly, cells were grown on 35 mm MatTek glass-bottom dishes (density, 2 × 10^5^ cells/well). MG-132 was used as a positive control. After treatment, cells were washed (3X) with PBS and fixed for 15 min with 4% (v/v) paraformaldehyde (pH 7.4) at room temperature. Cells were washed again (3X) with PBS, permeabilized with permeabilizing solution, and gently shaken on ice for 30 min. Next, the cells were washed (2X) with PBS and incubated with the Dual Detection Reagent for 30 min at room temperature. Subsequently, the cells were washed with PBS, and fluorescent images were captured using the 63x oil objective by confocal microscopy (Leica TCS SP5 II confocal microscope, Wetzlar, Germany).

### Biotin switch assay

S-NO proteins were identified in HUVECs and carotid sections by replacing S-nitrosyl groups with biotin using the S-NO protein detection assay kit (Cayman Chemical Co, catalog no.: 10006518) according to the manufacturer's instructions and observed with confocal microscopy. Biotinylated proteins were also detected by Western immunoblotting using Avidin-HRP or purified using Streptavidin Agarose Resin (Thermo Scientific, #20347) by incubating samples with the matrix for 2 h at 4°C under stirring. After washing with Tris-buffered saline with Tween-20, purified proteins were eluted with Protein Extraction Reagent Type 4 (P4, Sigma-Aldrich) supplemented with 30 mM biotin adjusted to pH 14, under stirring overnight at room temperature. Purified proteins were analyzed by MS.

### Proteomic analysis

Proteins recovered from the purification step were processed by the EasyPep MS Sample Kit (Thermo Scientific). Eluted peptides were dried with a SpeedVac vacuum centrifuge (Savant-SPD121P). Prior to MS analysis, peptides were solubilized in 0.1% formic acid, and the peptide content was quantified through the BCA quantitative colorimetric peptide assay (Thermo Fisher Scientific). Each sample (1.9 μg) was injected (in quintuplicate) into an UltiMate 3000 RSLC nano system coupled to the Exploris 240 mass spectrometer (Thermo Fisher Scientific) and resolved by Easy-Spray Pepmap RSLC 18 (2 μm, 50 cm × 75 μm) at a flow rate of 250 nl/min with a gradient of phase B (80% acetonitrile/0.1% formic acid); solvent A was 0.1% formic acid in water, from 2% to 40% in 130 min. Phase B was then changed up 99% in 40 min, kept for 5 min, and then the column was re-equilibrated for 10 min.

Mass spectra were acquired in a positive mode and data-dependent manner. For MS1, the *m/z* range was set to 350–1,500 at 120,000 resolution (at *m/z* 200), automatic gain control target 3e6, and auto maximum injection time. MS2 switched when ion intensity was above 5e3, with an *m/z* range in auto mode, normalized high-energy collisional dissociation (HCD) energy of 30%, automatic gain control target of 7.5e4, and maximum injection time of 40 ms. The resolution was set to 15,000 at *m/z* 200, and the internal calibrant was employed in run start mode. Raw data generated by Xcalibur 4.2 software (Thermo Fisher Scientific) were analyzed using Proteome Discoverer 2.5 (Thermo Fisher Scientific) by using the MSPepSearch algorithm: ProteomeTools_HCD30_PD and NIST_Human_Orbitrap_HCD databases were employed. After false discovery rate (FDR) evaluation by a target-decoy strategy in concatenated *q*-value mode, spectra with an FDR lower than 0.01 were sent to the Sequest HT algorithm. It was considered the fixed modification cysteine carbamidomethylation and the variable serine, threonine, or tyrosine phosphorylation. Also, variable oxidation (M) and deamidation (N, Q) were considered. The FDR was again evaluated by a target-decoy strategy in a concatenated *q*-value manner. FDR (strict) was set as 0.01, whereas FDR (relaxed) was set as 0.05. Differentially expressed and exclusive proteins were evaluated. A *t*-test background-based analysis was performed for proteomic data.

### Subcellular fractionation

In order to separate the subcellular organelles, an isopycnic sucrose gradient was performed by ultracentrifugation as described (22). After treatment, 3.5 × 10^6^ cells were homogenized with a buffer without detergent (12% sucrose, 10 mM KCl, 2 mM MgCl_2_, 100 mM Tris-HCl, pH 7.8). The homogenate (600 μl) was loaded on top of a 16–55% (w/w) continuous sucrose gradient. After centrifugation at 141,000*g* for 4 h at 4°C in a Beckman SW28 rotor (Beckman Coulter), fractions of 850 μl were collected, and the total proteins present were precipitated with 150 μl of trichloroacetic acid per sample, centrifuged at 12,000 rpm, and washed twice with acetone. The pellets obtained were resuspended with loading buffer and then analyzed by SDS-PAGE and immunoblotting with the anti-BiP (1:2,000 dilution; Euroclone, anti-BiP/Grp78 [C50B12] Rabbit mAb) and anti-lysosome-associated membrane protein 1 (LAMP1) (1:2,000 dilution; Cell Signaling Technology, rabbit mAb clone C54H11, catalog no.: 3243).

### Statistical analysis

Statistical analyses were performed using Prism, version 9.00 (GraphPad Software). Data are presented as the mean ± SD from at least three independent biological experiments. Differences between samples were assessed by ANOVA with Tukey post hoc analysis. The *t-*test was utilized for data comparison between two groups. The *P* value was calculated using a two-tailed test, where the value of *P* < 0.05 was indicative of a statistically significant difference.

## Results

### SEC-B affects ER structure and triggers the UPR activation

**Fig. 1:**
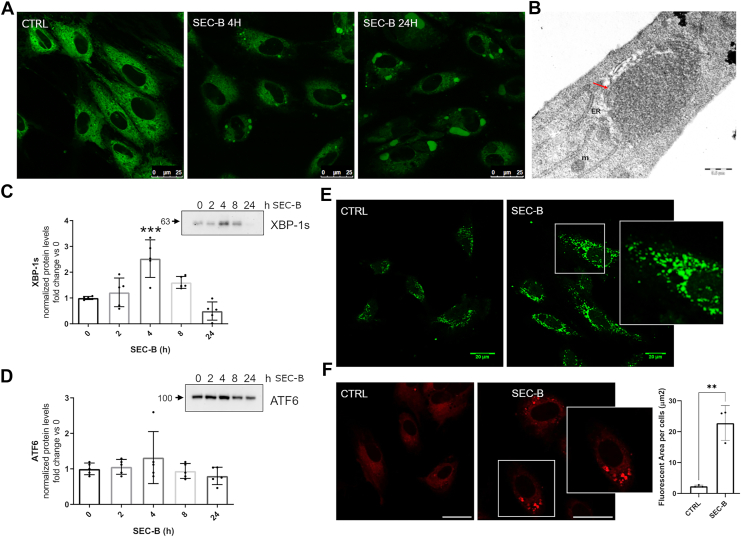
SEC-B induces UPR activation, leading to ER stress. A: Confocal microscopy of ER-Tracker Green-stained HUVECs after 4 and 24 h of treatment with 10 μM SEC-B. The scale bar represents 25 μm. B: TEM images also showed the disorganization and enlargement of ER membranes as indicated by the red arrow (the scale bar represents 0.5 μm). C and D: Western immunoblotting analysis and quantification of XBP-1s and ATF6 expression levels in HUVECs treated with 10 μM SEC-B. Values are the mean ± SD of the values obtained from five independent experiments. ∗∗∗*P* < 0.001 versus untreated cells. E: Confocal images of GRP78 immunostaining in HUVECs after 24 h treatment with 10 μM SEC-B. The scale bar represents 20 μm. F: Aggresome formation detected by the ProteoStat® Aggresome Detection Kit after 24 h of 10 μM SEC-B treatment. The scale bar represents 25 μm. Quantitative analysis of positive fluorescent area per cell, in HUVECs treated with 10 μM SEC-B for 24 h. Data are expressed as mean ± SD (n = 3). ∗∗*P* < 0.01 versus untreated cells.

To investigate the effects of SEC-B on the ER, we initially examined ER structure by fluorescence microscopy in treated HUVECs. As shown in Fig. 1A, SEC-B induces marked disorganization and enlargement of ER membranes. This alteration was detectable after 4 h of treatment but became more pronounced at 24 h. Transmission electron microscopy images (Fig. 1B) confirmed structural alterations in the ER, as it appeared dilated and highly disorganized, scattered throughout the cytoplasm following treatment. ER alteration was also studied from a functional point of view by analyzing activation of key stress-sensor pathways of the UPR, specifically XBP-1 and ATF6, which have been involved in mediating ER expansion (23, 24). Our results show that SEC-B promotes a significant increase in XBP-1s expression, by approximately 2-fold, at 4 h of treatment, whereas a slight modulation of ATF6 was observed (Fig. 1C, D). During ER stress, GRP78 is upregulated and can relocate to other cellular compartments where it acquires new functions (25). Immunofluorescence analysis revealed a specific redistribution of GRP78 in the cytoplasm after 24 h treatment (Fig. 1E). Accumulation of misfolded proteins has been previously associated with prolonged ER stress (26). The staining with the aggresome detection kit revealed a red punctate pattern in the cytoplasm, highlighting an increased accumulation of misfolded proteins in SEC-B-treated cells (Fig. 1F).

### SEC-B induces NSS by increasing NO/inducible nitric oxide synthase expression in HUVECs

**Fig. 2:**
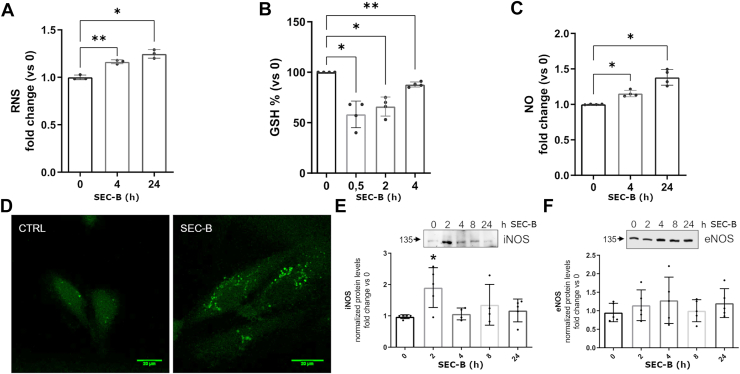
SEC-B induces NSS through increased intracellular NO and RNS and depletion of the antioxidant GSH. Quantitative analysis of RNS (A), GSH (B), and NO (C) levels in HUVECs treated with 10 μM SEC-B. Data are expressed as the mean ± SD of the values obtained from at least three independent experiments.∗*P* < 0.05, ∗∗*P* < 0.01 versus untreated cells. D: Confocal microscopy of 4,5-diaminofluorescein diacetate-stained HUVECs after 24 h of treatment with 10 μM SEC-B. The scale bar represents 20 μm. E and F: Western immunoblotting analysis and quantitation of iNOS and eNOS expression levels in HUVECs treated with 10 μM SEC-B. Data are reported in the graph as fold change versus untreated cells. Data are expressed as the mean ± SD of the values obtained from at least four independent experiments. ∗*P* < 0.05 versus untreated cells.

To further elucidate the mechanism by which SEC-B promotes ER stress, we assessed redox perturbation following treatment. Intracellular levels of RNS, GSH, and NO were measured in HUVECs. As shown in Figure 2A, B, SEC-B significantly increased RNS levels during 24 h of treatment and decreased GSH content within the first 4 h. In parallel, NO production increased over 24 h, as confirmed by confocal microscopy images showing a different pattern of fluorescence (Fig. 2C, D). Quantification of NO synthase (NOS) enzymes highlighted an early increase of inducible nitric oxide synthase (iNOS) protein levels by 2-fold, whereas the constitutive eNOS remained unchanged during the treatment (Fig. 2E, F).

### SEC-B increases total protein S-NO in HUVECs, including GRP78 and PDIA6

**Fig. 3:**
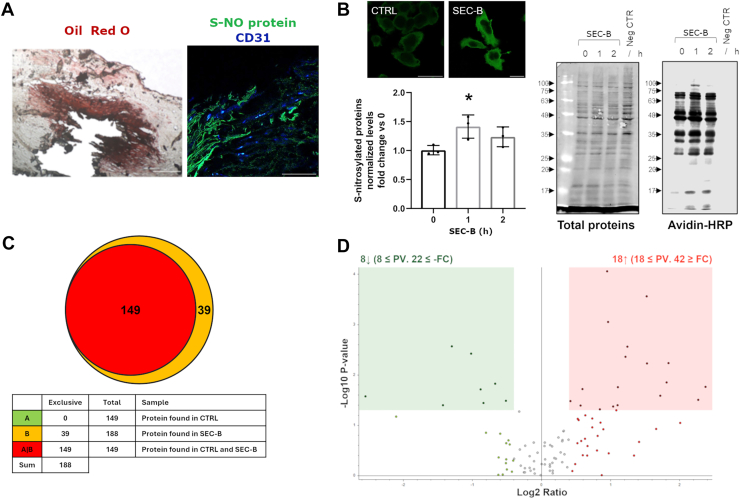
SEC-B promotes protein S-NO in HUVECs. A: Accumulation of lipids was observed by staining with Oil Red O. Confocal images of CD31 immunostaining (blue) and S-NO proteins (green) in carotid sections of patients with advanced atherosclerosis. The scale bar represents 50 μm. B: Confocal images of S-NO proteins in HUVECs treated with 10 μM SEC-B for 2 h. The scale bar represents 50 μm. Quantitative analysis of S-NO protein levels in HUVECs treated with 10 μM SEC-B, performed by Western blot analysis using Avidin-HRP detection. A representative image is shown on the right, while the graph shows the quantification of three independent experiments. Data are expressed as the mean ± SD. ∗*P* < 0.05 versus untreated cells. Total proteins were stained as the loading control. C: Venn diagram of differentially S-NO proteins in SEC-B-treated cells compared to untreated control cells. D: The volcano plot shows significantly hyper-S-nitrosylated (in red) and hypo-S-nitrosylated (in green) proteins. Proteins are selected with a log2 fold change of 0.4 and an unadjusted *P* < 0.05.

Aberrant S-NO of proteins is a feature observed in various pathological conditions associated with NSS (18, 27). Our investigation performed on carotid sections from patients with advanced atherosclerosis confirmed the massive lipid accumulation in the lesion area and detected the presence of S-NO proteins in the altered endothelial layer (Fig. 3A). Accumulation of S-NO proteins was also observed in HUVECs by confocal microscopy (Fig. 3B). Western immunoblotting analysis revealed a significant increase in S-NO protein content after 1 h of SEC-B treatment (Fig. 3B). To identify S-NO proteins, cell extracts were enriched for S-NO proteins by Streptavidin Agarose Resin and analyzed by MS. MS/MS data analysis allowed us to identify a total of 188 master proteins, of which 149 were expressed both in Ctrl and SEC-B groups, whereas 39 were exclusive to SEC-B treatment (Fig. 3C and supplemental Table S1). Interestingly, among exclusive S-NO proteins of SEC-B-treated samples, we identified protein disulfide isomerase family A member 6 (PDIA6), which is involved in limiting UPR signaling (28). Moreover, among coexpressed proteins, 26 proteins were found differentially S-nitrosylated between Ctrl and SEC-B groups, 18 were hyper-S-nitrosylated and 8 proteins were hypo-S-nitrosylated after treatment (Fig. 3D and Table 1). Notably, among the hyper-S-NO proteins, we identified GRP78, whose overall levels in whole cell extracts remained unchanged, suggesting that SEC-B increases the fraction of S-NO GRP78 rather than its synthesis (supplemental Fig. S1).

**Table 1:** Differentially S-NO proteins in SEC-B-treated HUVECs

Accession	Description	Gene symbol	Abundance ratio	Abundance ratio adjusted *P* value
Hyper-S-nitrosylated				
P81605	Dermcidin	DCD	2.373	6.26E-02
P13639	Elongation factor 2	EEF2	1.510	6.26E-02
E9PQB7	Cofilin-1	CFL1	1.480	6.38E-02
Q96DR8	Mucin-like protein 1	MUCL1	1.954	6.26E-02
V9HWB4	78 kDa glucose-regulated protein	HEL-S-89n	1.936	6.04E-02
P48681	Nestin	NES	3.512	6.26E-02
Q9NZT1	Calmodulin-like protein 5	CALML5	1.340	6.26E-02
Q15657	Tropomyosin isoform		2.156	6.26E-02
P12273	Prolactin-inducible protein	PIP	5.184	6.26E-02
V9HWC0	Moesin	HEL70	2.150	6.26E-02
B7Z1K5	Tubulin alpha chain		3.302	6.26E-02
E9PKE3	Heat shock cognate 71 kDa protein	HSPA8	1.795	7.54E-02
A0A7I2V599	60 kDa heat shock protein, mitochondrial	HSPD1	2.890	6.26E-02
A0A0U1RQZ6	Zymogen granule protein 16 homolog B	ZG16B	2.877	6.04E-02
C9JF17	Apolipoprotein D	APOD	2.329	6.26E-02
H7C144	Alpha-actinin-4	ACTN4	2.053	6.61E-02
A0A060VCY6	MHC class I antigen	HLA-A	4.828	6.26E-02
A1A4E9	Keratin 13	KRT13	3.581	6.26E-02
Hypo-S-nitrosylated				
Q5T749	Keratinocyte proline-rich protein	KPRP	0.495	6.26E-02
P78371	T-complex protein 1 subunit beta	CCT2	0.372	6.28E-02
B4DUQ1	Heterogeneous nuclear ribonucleoprotein K	HNRNPK	0.171	6.26E-02
Q59EM6	Internexin neuronal intermediate filament protein, alpha variant		0.629	6.26E-02
Q53ET2	Dihydropyrimidinase-related protein 2		0.543	6.26E-02
Q16778	Histone H2B type 2-E	H2BC21	0.408	6.26E-02
P50991	T-complex protein 1 subunit delta	CCT4	0.559	6.26E-02
P08670	Vimentin	VIM	0.700	6.26E-02

The table shows significantly hyper-S-NO and hypo-S-NO proteins; data are obtained by Proteome Discoverer software from SEC-B-treated versus untreated Ctrl cells. Proteins are selected with log2 fold change 0.4 and adjusted *P* < 0.1.

### NOS inhibition reverses ER dysfunction and prevents aggresome formation

**Fig. 4:**
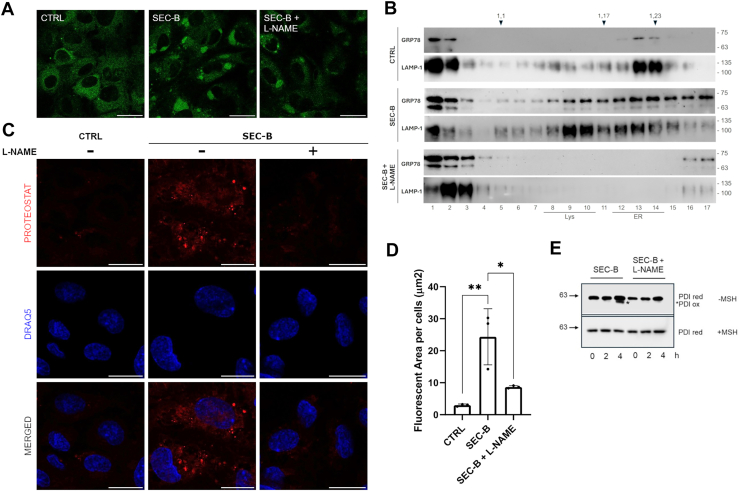
NOS inhibitor reduces ER swelling and aggresome formation. A: Confocal microscopy of ER-Tracker Green-stained HUVECs after 24 h of treatment with 10 μM SEC-B with and without L-NAME pretreatment. The scale bar represents 25 μm. B: Distribution of the GRP78 and LAMP1 protein in HUVECs. Total proteins extracted from HUVECs treated with SEC-B 10 μM or pretreated with L-NAME before SEC-B treatment were fractionated by centrifugation on an isopycnic sucrose gradient. Total proteins from each fraction were analyzed by SDS-PAGE and immunoblot using anti-GRP78 or anti-LAMP1 antiserum. The top of the gradients is at the left; numbers on the top indicate density (g/ml); numbers on the bottom indicate the gradient fractions. The lysosomal and ER fractions are underlined. The positions of molecular mass markers in kilodaltons are indicated by numbers on the right of the panels. C: Aggresome formation detected by the ProteoStat® Aggresome Detection Kit after 24 h of treatment with 10 μM SEC-B with and without L-NAME pretreatment. The scale bar represents 25 μm. D: Quantitative analysis of positive fluorescent area per cell, in HUVECs treated with 10 μM SEC-B with and without L-NAME pretreatment. Data are expressed as mean ± SD (n = 3). ∗∗*P* < 0.01 versus untreated cells; ∗*P* < 0.05 versus SEC-B-treated cells. E: Immunoblot analysis of PDI expression in lysates of HUVECs treated with 10 μM SEC-B with and without L-NAME. Proteins were resolved under reducing (+MSH) and nonreducing (-MSH) conditions. An asterisk indicates oxidized PDI.

To investigate the role of nitrosylation in ER stress, HUVECs were pretreated with the NOS inhibitor, L-NAME, which selectively reversed iNOS expression and reduced NO production in HUVECs (supplemental Fig. S2). Interestingly, we found that L-NAME is able to reduce ER swelling as observed by microscopy analysis in Figure 4A.

To quantify ER alterations, an isopycnic gradient centrifugation of cell lysates was performed. Western blotting was performed using antibodies recognizing GRP78 and the lysosomal membrane marker LAMP1, followed by analysis of the obtained fractions. A different distribution of GRP78 is observed in cells treated with SEC-B, where it concentrates in aggresomes that precipitate into higher-density fractions. Conversely, pretreatment with L-NAME was able to restore the amount of GRP78 to basal levels and eliminate the presence of protein aggregates (Fig. 4B). Furthermore, the data obtained revealed the colocalization in the same fractions of the soluble ER resident protein GRP78 with LAMP1. The two proteins were present both in the low-density fractions (1–3), corresponding to soluble proteins derived from the cytosol and proteins released from organelles during cell rupture treatment, and in the denser fractions (8–11 lysosome and 12–15 ER). This finding suggests that the large aggregates present in samples treated with SEC-B, visualized by confocal microscopy (Fig. 1F), are probably autophagic structures that degrade parts of the ER overloaded with misfolded proteins bound to the chaperone GRP78. Furthermore, as shown in Figure 4C, D, L-NAME treatment led to a sharp decrease (approximately 60%; *P* < 0.05) of misfolded protein aggregates. Western blot analysis of PDI under nonreducing conditions showed that PDI was mainly reduced in control cells, whereas SEC-B promoted partial PDI oxidation as determined by the appearance of a second immunoreactive band with increased mobility at 4 h of treatment (29). This band disappeared under reducing conditions, indicating that the shift was due to cysteine oxidation. Cell pretreatment with L-NAME completely abrogated PDI oxidation, indicating that aberrant NO production contributes to PDI redox imbalance, thereby compromising its enzymatic activity in catalyzing disulfide bond formation and isomerization (Fig. 4E).

## Discussion

The ER is involved in many essential cellular processes, including protein folding and quality control of newly synthesized proteins (30). The mechanisms that promote ER stress are involved in the pathogenesis of several disorders, such as neurodegenerative diseases and atherosclerosis. Our previous findings demonstrated that SEC-B treatment induced ER stress in HUVECs, as evidenced by significant changes in ER size and shape (8). Additionally, SEC-B rapidly activates microvascular endothelial cells by inducing oxidative stress, NO production, and proinflammatory cytokine release (20). Considering these findings, we investigated the molecular mechanisms involved in SEC-B-induced ER stress.

Our data confirmed that noncytotoxic doses of SEC-B caused marked ER swelling. This was preceded by activation of the IRE/XBP-1 branch of the UPR and was associated with accumulation of misfolded proteins at 24 h post-treatment.

Growing evidence indicates that XBP-1s overexpression activates phospholipid biosynthesis and ER biogenesis, which are compensatory mechanisms to alleviate ER stress, independently of an increase in ER chaperone levels (31, 32). Furthermore, the ATF6 and the IRE1α/XBP-1 cascades transcriptionally upregulate ER chaperone genes to promote efficient folding and degradation of proteins, facilitating efficient ER function (33). Conversely, ER expansion and aggresome formation are both consequences of ER stress triggered by the accumulation of misfolded proteins. In particular, aggresomes play a protective role by sequestering protein aggregates and sustaining ER homeostasis (34).

However, if the efficiency of the aggresome degradation by proteasomes and the autophagic pathway is impaired, the risk of pathogenic outcomes rises (35).

NO acts as a key signaling molecule that, within normal physiological levels, regulates several biological processes like vasodilation, immune responses, and neurotransmission. However, an excessive presence of NO becomes cytotoxic to intracellular components, resulting in severe damage to intracellular lipids, proteins, enzymes, and other molecules, and contributing to the development of various diseases (36). The iNOS is not present in resting cells but can be induced in response to inflammatory stimuli, producing a large amount of NO often accompanied by increased ROS production, including peroxynitrite and superoxide, which contribute to plaque instability (37).

Our findings demonstrate that SEC-B induces iNOS expression and increases cellular NO and RNS levels, coupled with early depletion of GSH, suggesting that SEC-B triggers NSS in endothelial cells. Similarly, Wielkoszyński *et al.* (38) showed that 5α,6α-epoxyphytosterols exacerbate NSS and inflammatory cytokine release and impair lipid metabolism in rats on low-cholesterol diets. Recently, Su *et al.* (39) showed that oxLDL strongly upregulates inflammatory mediators like iNOS and suppresses eNOS activity, thereby contributing to endothelial dysfunction in mouse and rat artery endothelial cells.

One of the key mechanisms by which NO regulates the function and the activity of various target proteins is a process commonly known as S-NO, a post-translational protein modification in which NO covalently binds to cysteine thiols (17). While tightly regulated S-NO is essential in multiple biological processes, including transcription, DNA repair, cell growth/differentiation, and apoptosis, its dysregulation is implicated in various diseases (40). The results demonstrate that hypo- or hyper-S-NO of specific protein targets is involved in the development and progression of several diseases (26, 41, 42).

Chao *et al.* (43) reported that increased protein nitrosylation of specific proteins, that is, the guanine nucleotide-binding protein 2 in the aorta of diabetic mice, accelerates atherosclerosis progression. Moreover, S-NO has been visualized in human blood vessel sections from patients with cardiovascular diseases and severe endothelial injury, as highlighted by our investigation performed on carotids from atherosclerotic patients (44, 45).

The overproduction of intracellular NO is closely associated with ER dysfunction, leading to ER stress-mediated apoptosis (15, 46). Nakato *et al.* (17) showed that S-NO of ER stress sensors, such as PERK and IRE, contributes to neurodegeneration by promoting ER stress, mitochondrial dysfunction, and synaptic degeneration. While altered protein S-NO has been demonstrated to promote dysfunctional ER stress signaling contributing to neuronal disease, this relationship needs further investigation in the context of the vascular system (47).

Based on this evidence, we explored the impact of SEC-B on protein S-NO. Our results confirmed that SEC-B significantly increases total protein S-NO in endothelial cells. This aligns with the findings of Wang *et al.* (48), who showed that iNOS-induced S-NO of eNOS at Cys94 and Cys99 exacerbates endothelial dysfunction under oxLDL exposure.

Among proteins specifically nitrosylated by SEC-B, we identified PDIA6, which belongs to the protein disulfide isomerase family and is essential for the formation, cleavage, and reorganization of disulfide bonds in proteins (28). PDIA6 is known to modulate the PERK pathway; therefore, PDIA6 deficiency leads to persistent activation of UPR signaling (49). Notably, S-NO of PDIA6 has been shown in Alzheimer's disease samples, indicating possible dysfunction of redox protein folding and UPR regulation in the disease etiology (49).

We also detected GRP78 among differentially expressed S-NO proteins. Together with PDI, GRP78 is a major ER chaperone involved in protein folding and ER-associated degradation and is known to undergo redox-sensitive post-translational modification. It has been demonstrated that cysteine oxidation alters GRP78 activity, impairing ATPase activity and enhancing substrate binding (50). Xu *et al.* (51) demonstrated that abnormal protein S-NO of the ER is one of the pathways implicated in endothelial dysfunction induced by oxLDL. Furthermore, Wadham *et al.* (52) reported that high glucose treatment decreases S-NO of GRP78 in endothelial cells, contributing to vascular complications in diabetes.

To further investigate the interplay between ER stress and S-NO, we treated HUVECs with L-NAME, a specific NOS inhibitor. Our finding showed that L-NAME mitigated ER swelling and significantly reduced aggresome formation indicating a specific role of NO in the ER homeostasis. We also observed colocalization of GRP78 with LAMP1, indicating that lysosomal degradation of both native and misfolded ER proteins is critical for restoring ER homeostasis (53). Studies have demonstrated that failure of the proteasomal degradation system leads to autophagic clearance of the aggresomes, with lysosomes repositioning around them to facilitate degradation. This positioning may represent a specialized mechanism that enhances autophagic degradation of misfolded proteins accumulating within the aggresome (12, 54). Furthermore, L-NAME reduced PDI oxidation, likely due to reduced NSS. Molteni *et al.* (55) reported that extended oxidation of PDI accelerates disulfide bond formation, promoting the accumulation of high molecular weight protein aggregates in the cytosol.

Overall, our findings demonstrate that protein S-NO plays a pivotal role in promoting ER stress and protein aggresome formation in endothelial cells exposed to SEC-B. This mechanism may contribute to explaining the vascular dysfunction observed in atherosclerosis and highlights potential therapeutic targets to preserve endothelial integrity.

## Conclusion

Given the critical role of nitrosylation and ER stress in the pathogenesis of atherosclerosis, this study advances our understanding of the proatherogenic effect of oxysterols and offers new insights into disease mechanisms and potential therapeutic targets.

S-NO is a complex process that contributes to the development and progression of atherosclerosis by modulating endothelial function, inflammation, oxidative stress, and related cellular processes. Elucidating the specific protein targets and molecular pathways involved in S-NO could be crucial for developing effective therapeutic strategies against atherosclerosis to improve clinical outcomes.

## Data availability

The datasets generated and/or analyzed during the current study are available from the corresponding author on reasonable request.

## Supplemental data

This article contains supplemental data.

## Conflict of interest

The authors declare that they have no conflicts of interest with the contents of this article.

## References

[1] Iuliano L. (2011). Pathways of cholesterol oxidation via non-enzymatic mechanisms. Chem. Phys. Lipids.

[2] Testa G., Rossin D., Poli G., Biasi F., Leonarduzzi G. (2018). Implication of oxysterols in chronic inflammatory human diseases. Biochimie.

[3] Poli G., Biasi F., Leonarduzzi G. (2013). Oxysterols in the pathogenesis of major chronic diseases. Redox Biol..

[4] Brown A.J., Jessup W. (1999). Oxysterols and atherosclerosis. Atherosclerosis.

[5] Chang Y.H., Abdalla D.S.P., Sevanian A. (1997). Characterization of cholesterol oxidation products formed by oxidative modification of low density lipoprotein. Free Radic. Biol. Med..

[6] Miyoshi N. (2018). Biochemical properties of cholesterol aldehyde secosterol and its derivatives. J. Clin. Biochem. Nutr..

[7] Tomono S., Miyoshi N., Ito M., Higashi T., Ohshima H. (2011). A highly sensitive LC–ESI-MS/MS method for the quantification of cholesterol ozonolysis products secosterol-A and secosterol-B after derivatization with 2-hydrazino-1-methylpyridine. J. Chromatogr. B.

[8] Luchetti F., Crinelli R., Nasoni M.G., Cesarini E., Canonico B., Guidi L. (2019). Secosterol-B affects endoplasmic reticulum structure in endothelial cells. J. Steroid Biochem. Mol. Biol..

[9] Ni L., Yang L., Lin Y. (2024). Recent progress of endoplasmic reticulum stress in the mechanism of atherosclerosis. Front Cardiovasc. Med..

[10] Yang S., Wu M., Li X., Zhao R., Zhao Y., Liu L. (2020). Role of endoplasmic reticulum stress in atherosclerosis and its potential as a therapeutic target. Oxid Med. Cell Longev..

[11] Lau Y.S., Mustafa M.R., Choy K.W., Chan S.M.H., Potocnik S., Herbert T.P. (2018). 3′,4′-dihydroxyflavonol ameliorates endoplasmic reticulum stress-induced apoptosis and endothelial dysfunction in mice. Sci. Rep..

[12] Gao J., Li M., Qin S., Zhang T., Jiang S., Hu Y. (2016). Cytosolic PINK1 promotes the targeting of ubiquitinated proteins to the aggresome-autophagy pathway during proteasomal stress. Autophagy.

[13] Dong Y., Zhang M., Wang S., Liang B., Zhao Z., Liu C. (2010). Activation of AMP-Activated protein kinase inhibits oxidized LDL-Triggered endoplasmic reticulum stress in vivo. Diabetes.

[14] Yong K.-T., Pu L.-Y., Yong P.-B., Sheng T.-Y., Shui P.-Z., Guo Q.-Z. (2016). - role of PERK/eIF2α/CHOP endoplasmic reticulum stress pathway in oxidized low-density lipoprotein mediated induction of endothelial apoptosis. Biomed. Environ. Sci..

[15] Gotoh T., Mori M. (2006). Nitric oxide and endoplasmic reticulum stress. Arterioscler. Thromb. Vasc. Biol..

[16] Wang F., Zhang D., Zhang D., Li P., Gao Y. (2021). Mitochondrial protein translation: emerging roles and clinical significance in disease. Front Cell Dev. Biol..

[17] Nakamura T., Tu S., Akhtar M.W., Sunico C.R., Okamoto S., Lipton S.A. (2013). Aberrant protein S-Nitrosylation in neurodegenerative diseases. Neuron.

[18] Nakato R., Ohkubo Y., Konishi A., Shibata M., Kaneko Y., Iwawaki T. (2015). Regulation of the unfolded protein response via S-nitrosylation of sensors of endoplasmic reticulum stress. Sci. Rep..

[19] Wang K., Bermúdez E., Pryor W.A. (1993). The ozonation of cholesterol: separation and identification of 2,4-dinitrophenulhydrazine derivatization products of 3β-hydroxy-5-oxo-5,6-secocholestan-6-al. Steroids.

[20] Nasoni M.G., Benedetti S., Crinelli R., Palma F., Canonico B., Monittola F. (2022). 3β-Hydroxy-5β-hydroxy-B-norcholestane-6β-carboxaldehyde (SEC-B) induces proinflammatory activation of human endothelial cells associated with nitric oxide production and endothelial nitric oxide Synthase/Caveolin-1 dysregulation. Antioxidants.

[21] Fraternale A., De Angelis M., De Santis R., Amatore D., Masini S., Monittola F. (2023). Targeting SARS-CoV -2 by synthetic dual-acting thiol compounds that inhibit Spike/ACE2 interaction and viral protein production. FASEB J..

[22] De Marchis F., Colanero S., Klein E.M., Mainieri D., Prota V.M., Bellucci M. (2018). Expression of CLAVATA3 fusions indicates rapid intracellular processing and a role of ERAD. Plant Sci..

[23] Bommiasamy H., Back S.H., Fagone P., Lee K., Meshinchi S., Vink E. (2009). ATF6α induces XBP1-independent expansion of the endoplasmic reticulum. J. Cell Sci..

[24] Shaffer A.L., Shapiro-Shelef M., Iwakoshi N.N., Lee A.-H., Qian S.-B., Zhao H. (2004). XBP1, downstream of Blimp-1, expands the secretory apparatus and other organelles, and increases protein synthesis in plasma cell differentiation. Immunity.

[25] Chen J., Lynn E.G., Sharma H., Byun J.H., Kenyon V.A., Sahu K.K. (2024). Small molecules targeting GRP78 mitigate anti-GRP78 autoantibody–mediated tissue factor procoagulant activity in cultured endothelial cells. J. Thromb. Haemost..

[26] Yuan S., She D., Jiang S., Deng N., Peng J., Ma L. (2024). Endoplasmic reticulum stress and therapeutic strategies in metabolic, neurodegenerative diseases and cancer. Mol. Med..

[27] Foster M.W., Hess D.T., Stamler J.S. (2009). Protein S-nitrosylation in health and disease: a current perspective. Trends Mol. Med..

[28] Eletto D., Eletto D., Dersh D., Gidalevitz T., Argon Y. (2014). Protein disulfide isomerase A6 controls the decay of IRE1α signaling via disulfide-dependent Association. Mol. Cell.

[29] Kranz P., Neumann F., Wolf A., Classen F., Pompsch M., Ocklenburg T. (2017). PDI is an essential redox-sensitive activator of PERK during the unfolded protein response (UPR). Cell Death Dis..

[30] Gething M.-J., Sambrook J. (1992). Protein folding in the cell. Nature.

[31] Schuck S., Prinz W.A., Thorn K.S., Voss C., Walter P. (2009). Membrane expansion alleviates endoplasmic reticulum stress independently of the unfolded protein response. J. Cell Biol..

[32] Sriburi R., Jackowski S., Mori K., Brewer J.W. (2004). XBP1: a link between the unfolded protein response, lipid biosynthesis, and biogenesis of the endoplasmic reticulum. J. Cell Biol..

[33] Cunard R. (2017). Endoplasmic Reticulum stress, a driver or an innocent bystander in endothelial dysfunction associated with hypertension?. Curr. Hypertens. Rep..

[34] Granell S., Baldini G., Mohammad S., Nicolin V., Narducci P., Storrie B. (2008). Sequestration of mutated α1-Antitrypsin into inclusion bodies is a cell-protective mechanism to maintain endoplasmic reticulum function. Mol. Biol. Cell.

[35] Dobson C.M. (2003). Protein folding and misfolding. Nature.

[36] Wang X., Duan J., Gao H., Niu Z., Zhang C., Jiang Z. (2025). Imaging nitric oxide dynamics in endoplasmic reticulum stress with a tailor-made near-infrared fluorescence probe. Sens Actuators B Chem..

[37] Senderovic A., Galijasevic S. (2024). The role of inducible nitric oxide synthase in assessing the functional level of coronary artery lesions in chronic coronary syndrome. Cardiol. Res..

[38] Wielkoszyński T., Zalejska-Fiolka J., Strzelczyk J.K., Owczarek A.J., Cholewka A., Kokoszczyk K. (2020). 5 *α* ,6 *α* -Epoxyphytosterols and 5 *α* ,6 *α* -Epoxycholesterol increase nitrosative stress and inflammatory cytokine production in rats on low-cholesterol diet. Oxid Med. Cell Longev..

[39] Su Z., Wang J., Xiao C., Zhong W., Liu J., Liu X. (2024). Functional role of Ash2l in oxLDL induced endothelial dysfunction and atherosclerosis. Cell Mol. Life Sci..

[40] Fernando V., Zheng X., Walia Y., Sharma V., Letson J., Furuta S. (2019). S-Nitrosylation: an emerging paradigm of Redox Signaling. Antioxidants.

[41] Nakamura T., Prikhodko O.A., Pirie E., Nagar S., Akhtar M.W., Oh C.-K. (2015). Aberrant protein S-nitrosylation contributes to the pathophysiology of neurodegenerative diseases. Neurobiol. Dis..

[42] Tan C., Li Y., Huang X., Wei M., Huang Y., Tang Z. (2019). Extensive protein S-nitrosylation associated with human pancreatic ductal adenocarcinoma pathogenesis. Cell Death Dis..

[43] Chao M.-L., Luo S., Zhang C., Zhou X., Zhou M., Wang J. (2021). S-nitrosylation-mediated coupling of G-protein alpha-2 with CXCR5 induces Hippo/YAP-dependent diabetes-accelerated atherosclerosis. Nat. Commun..

[44] Chen Y., Zhao S., Wang Y., Li Y., Bai L., Liu R. (2014). Homocysteine reduces protein S-nitrosylation in endothelium. Int. J. Mol. Med..

[45] Pan L., Lin Z., Tang X., Tian J., Zheng Q., Jing J. (2020). S-Nitrosylation of Plastin-3 exacerbates thoracic aortic dissection Formation via endothelial barrier dysfunction. Arterioscler. Thromb. Vasc. Biol..

[46] Doutheil J., Althausen S., Treiman M., Paschen W. (2000). Effect of nitric oxide on endoplasmic reticulum calcium homeostasis, protein synthesis and energy metabolism. Cell Calcium..

[47] Nasoni M.G., Crinelli R., Iuliano L., Luchetti F. (2023). When nitrosative stress hits the endoplasmic reticulum: possible implications in oxLDL/oxysterols-induced endothelial dysfunction. Free Radic. Biol. Med..

[48] Wang W., Wang D., Kong C., Li S., Xie L., Lin Z. (2019). eNOS S-nitrosylation mediated OxLDL-induced endothelial dysfunction via increasing the interaction of eNOS with β-catenin. Biochim. Biophys. Acta BBA - Mol. Basis Dis..

[49] Medinas D.B., Rozas P., Hetz C. (2022). Critical roles of protein disulfide isomerases in balancing proteostasis in the nervous system. J. Biol. Chem..

[50] Ellgaard L., Sevier C.S., Bulleid N.J. (2018). How are proteins reduced in the endoplasmic reticulum?. Trends Biochem. Sci..

[51] Xu X., Qiu H., Shi F., Wang Z., Wang X., Jin L. (2019). The protein S-nitrosylation of splicing and translational machinery in vascular endothelial cells is susceptible to oxidative stress induced by oxidized low-density lipoprotein. J. Proteomics.

[52] Wadham C., Parker A., Wang L., Xia P. (2007). High glucose attenuates protein S-Nitrosylation in endothelial cells: role of oxidative stress. Diabetes.

[53] Hayashi Y., Ichijo H. (2024). Massive ER protein disposal by reticulophagy receptors and selective disposal by TOLLIP. Autophagy.

[54] Zaarur N., Meriin A.B., Bejarano E., Xu X., Gabai V.L., Cuervo A.M. (2014). Proteasome failure promotes positioning of lysosomes around the aggresome via local block of microtubule-dependent transport. Mol. Cell Biol..

[55] Molteni S.N., Fassio A., Ciriolo M.R., Filomeni G., Pasqualetto E., Fagioli C. (2004). Glutathione limits Ero1-dependent oxidation in the endoplasmic reticulum. J. Biol. Chem..

