# Supplementary material for: S-nitrosylation contributes to ER stress and aggresome formation in secosterol-B-mediated endothelial dysfunction
Source: J Lipid Res. 2026 Mar 10;67(4):101017. doi: 10.1016/j.jlr.2026.101017 (PMC13068821; doi:10.1016/j.jlr.2026.101017)

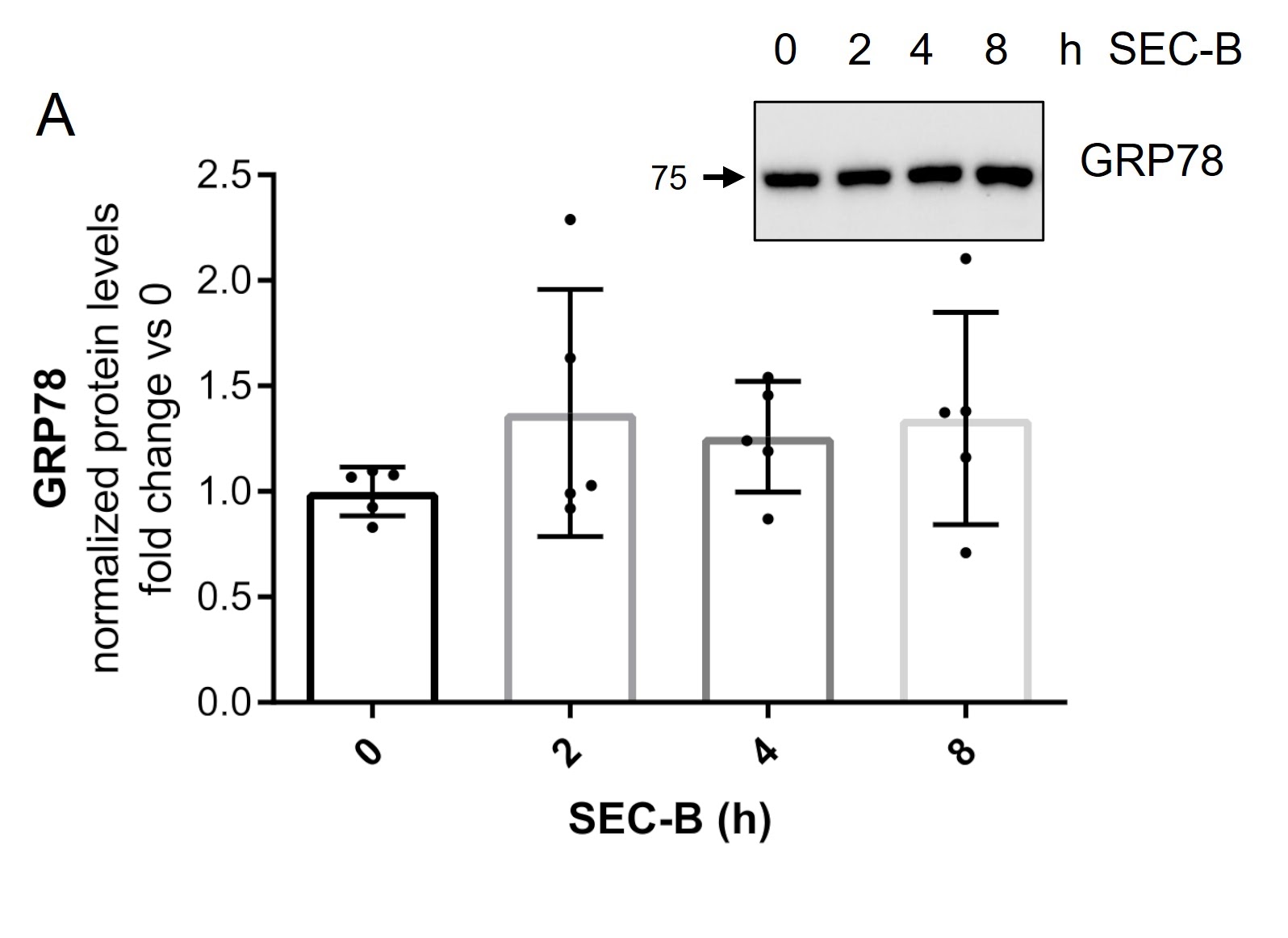


**Figure S1.** Quantitative analysis of GRP78 expression levels in HUVECs treated with 10 μM SEC-B. Data are expressed as the mean ± SD of the values obtained from 5 independent experiments.


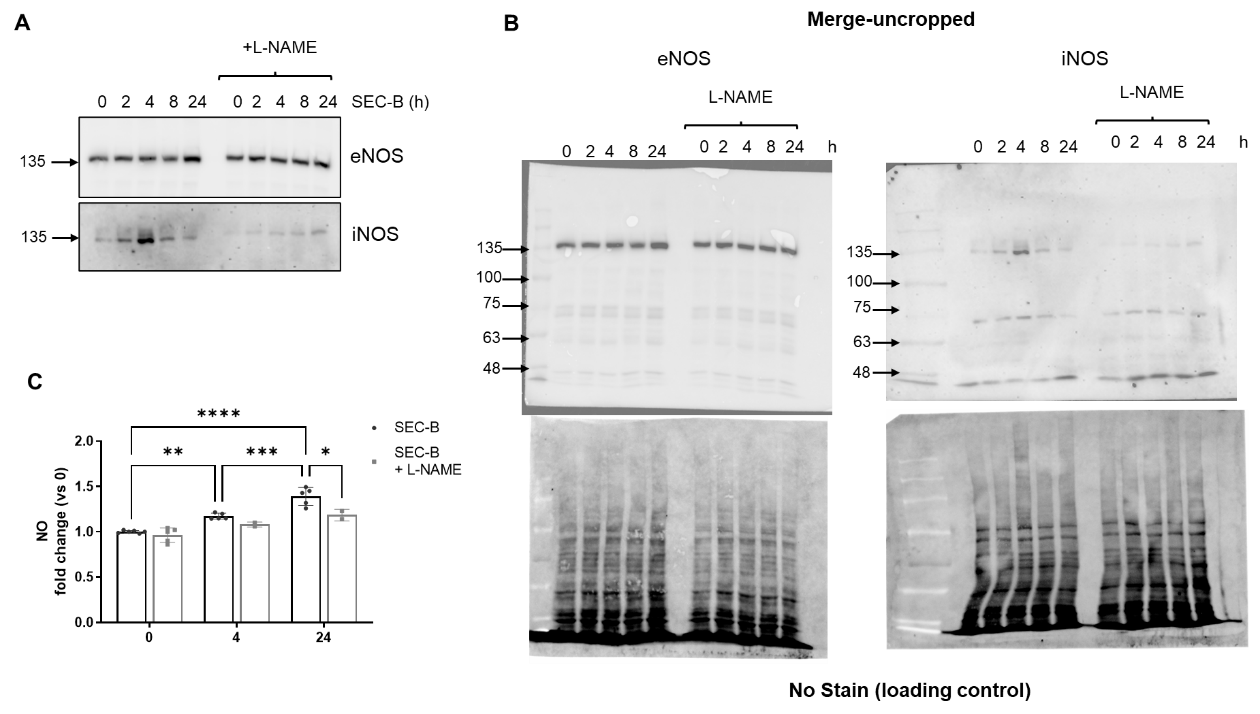


**Figure S2. L-NAME selectively reversed iNOS expression and reduced NO production.** **A)** Evaluation of iNOS and eNOS protein expression in HUVECs treated with 10 μM SEC-B and with L-NAME and relative merge-uncropped blots **B). C)** Quantification of intracellular NO in SEC-B-treated HUVECs with or without L-NAME.

**Supplementary Table S1.** Inferred data obtained by Proteome Discoverer software from SEC-B-treated *vs* untreated Ctrl cells; “log_2_ ratio” and “found in sample columns” are highlighted in yellow. In orange exclusive proteins, in red hyper-nitrosylated proteins, in green hypo-nitrosylated proteins.

**Uncropped blot**


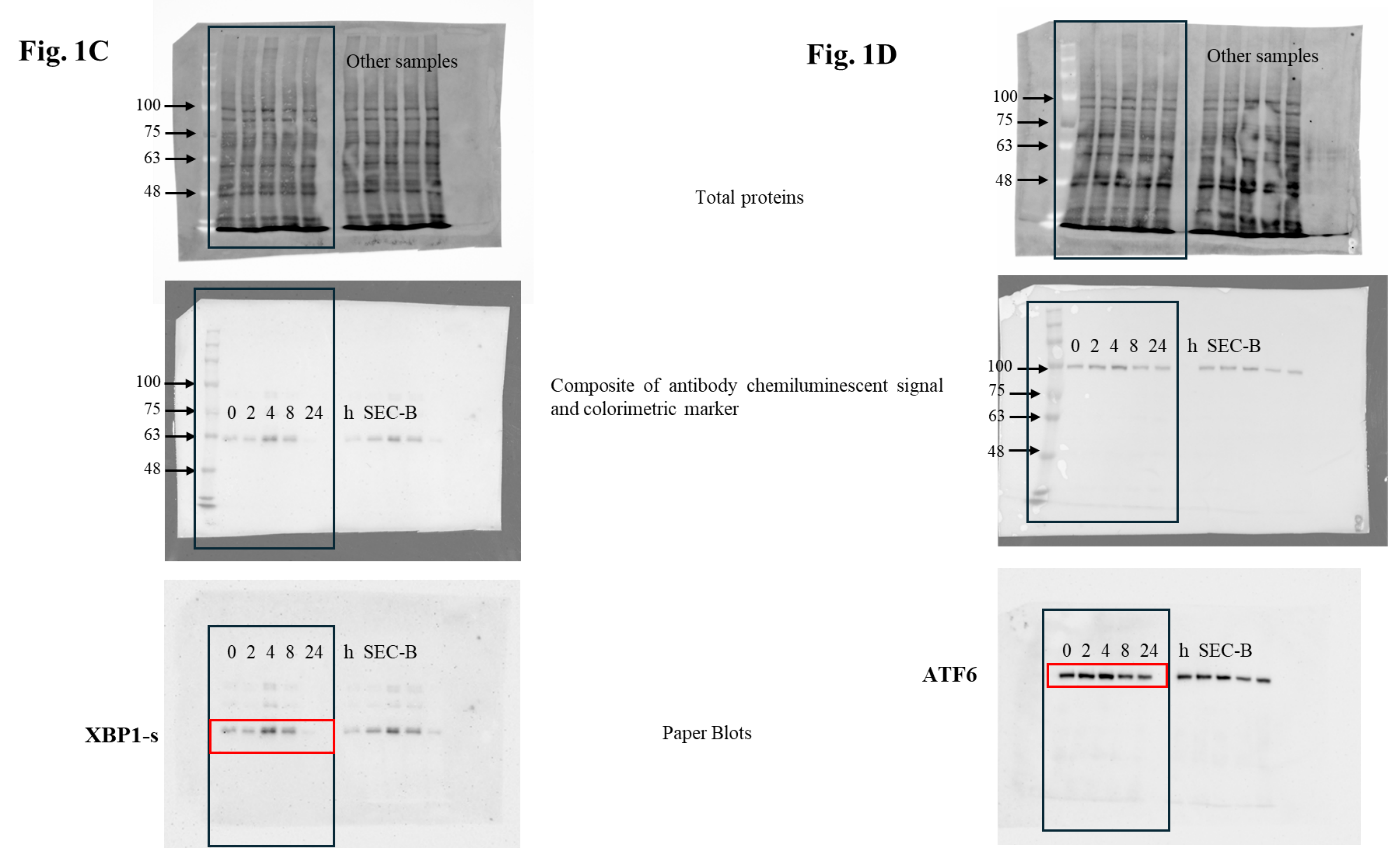


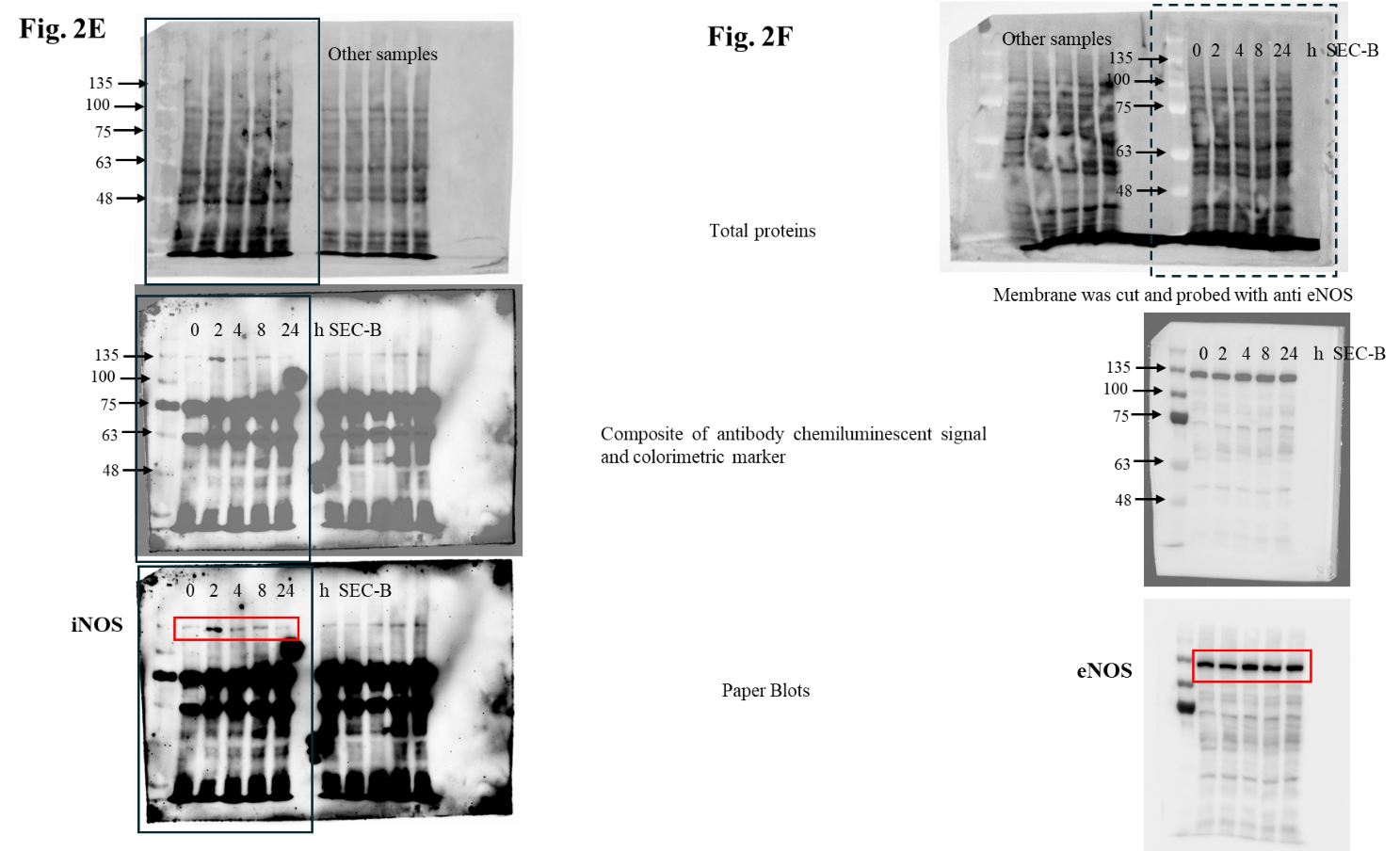


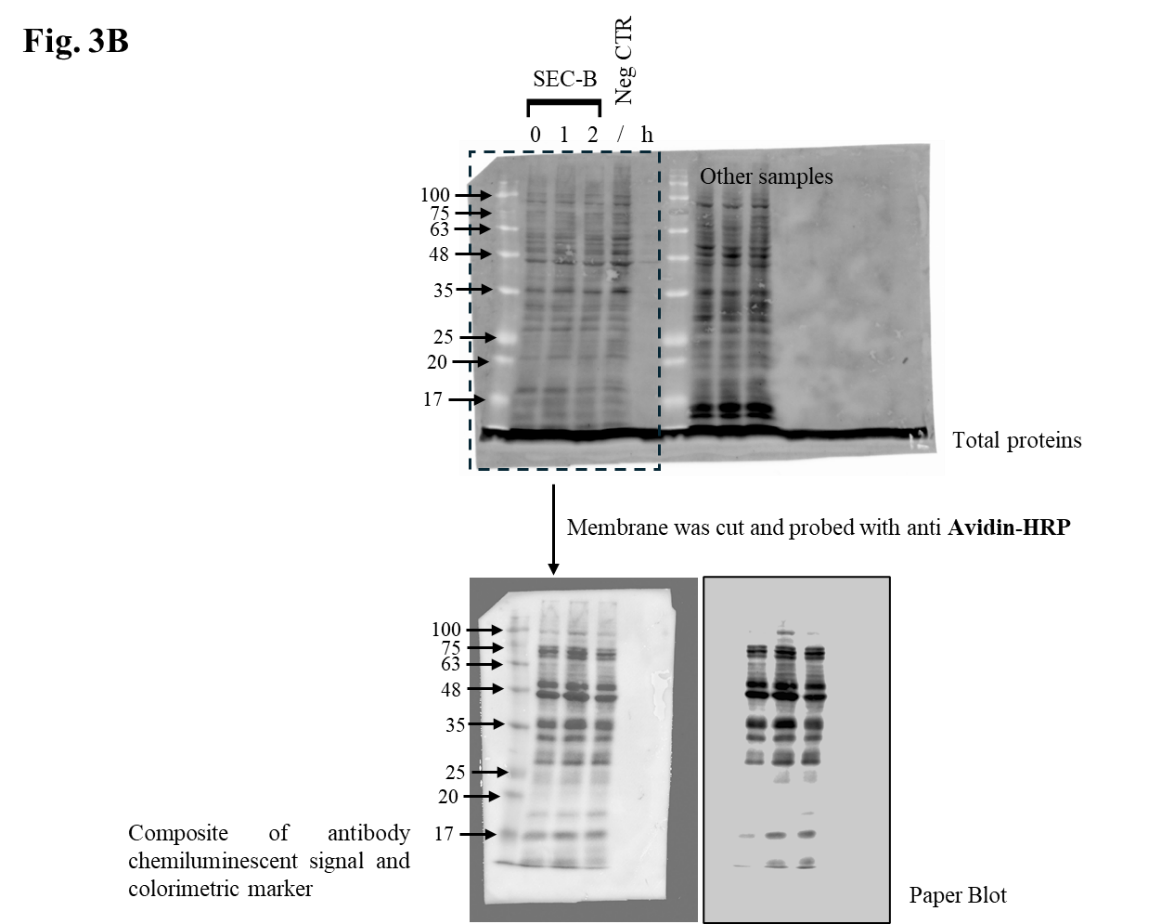


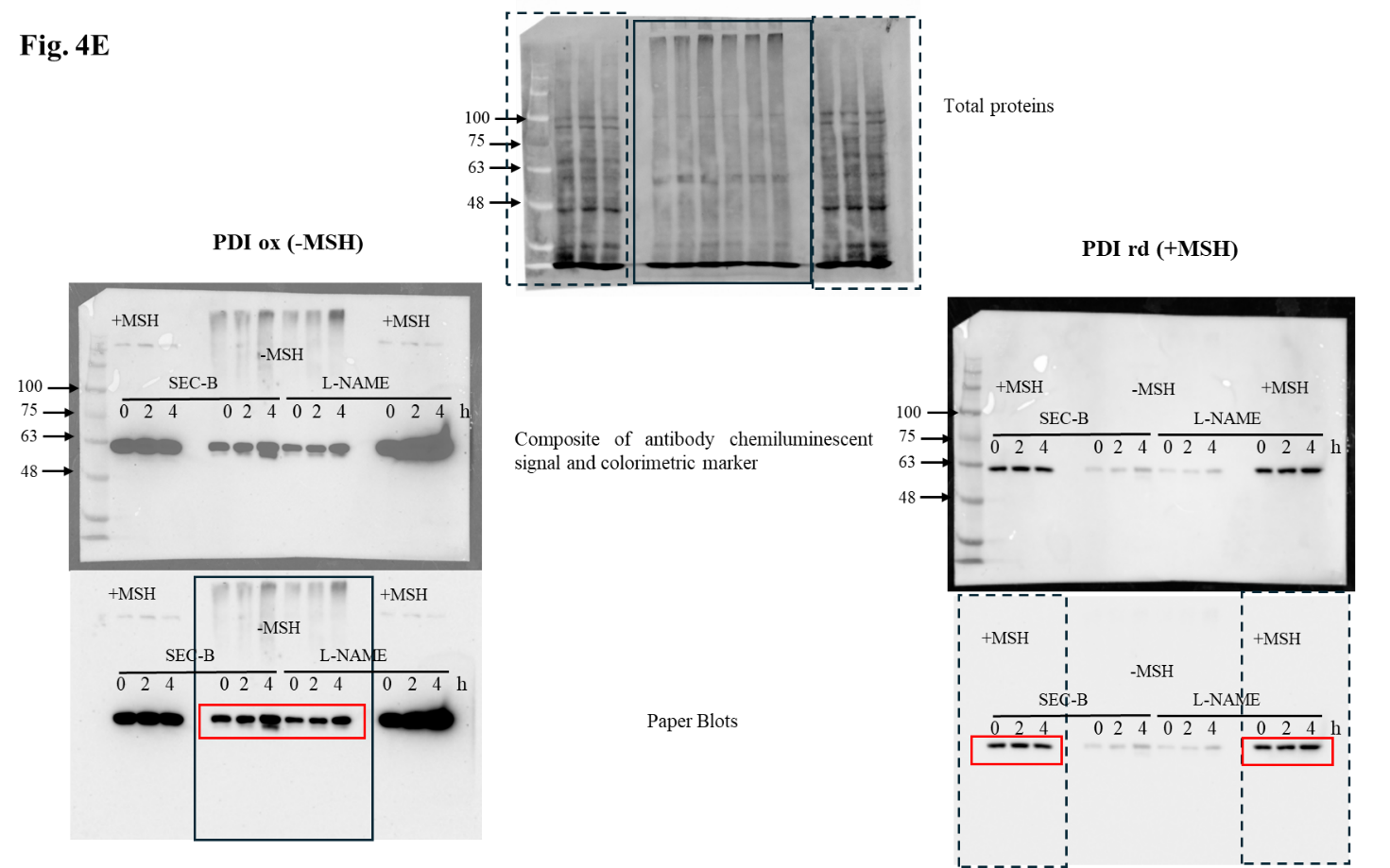


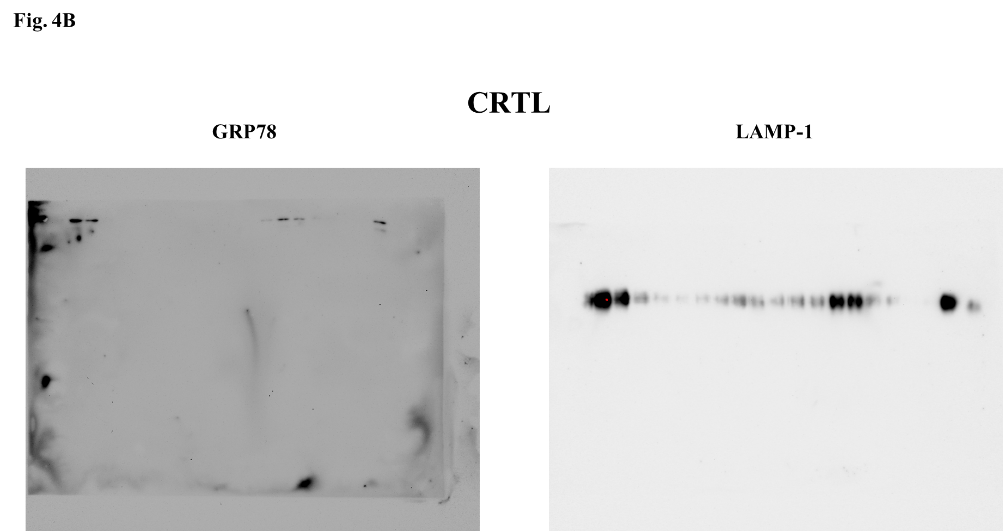


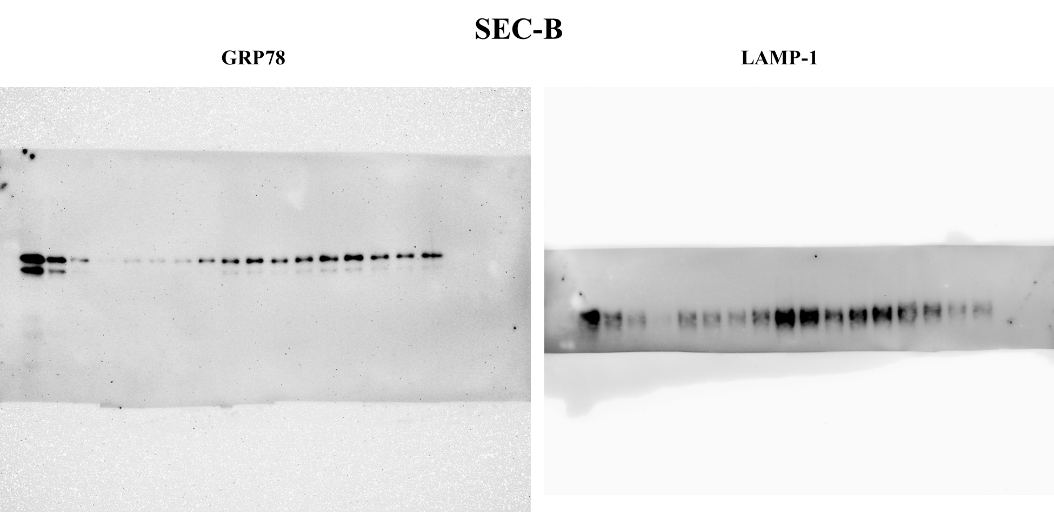


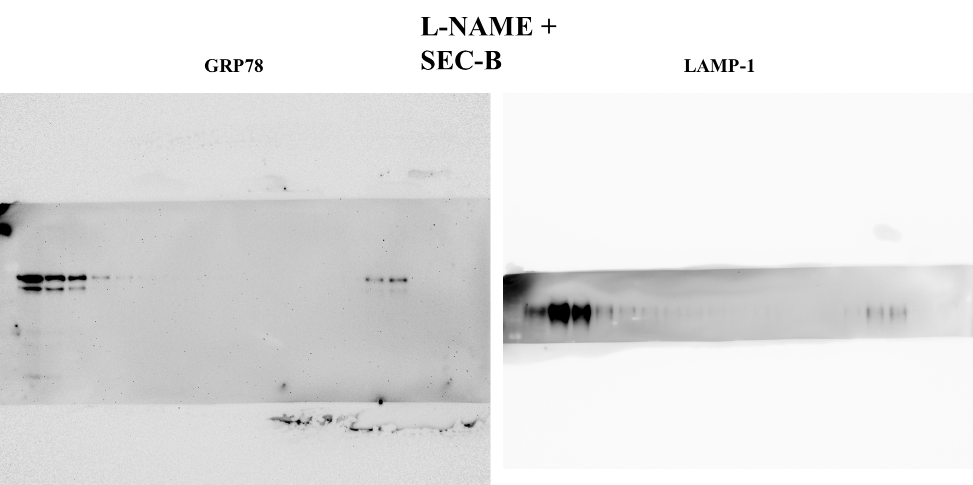


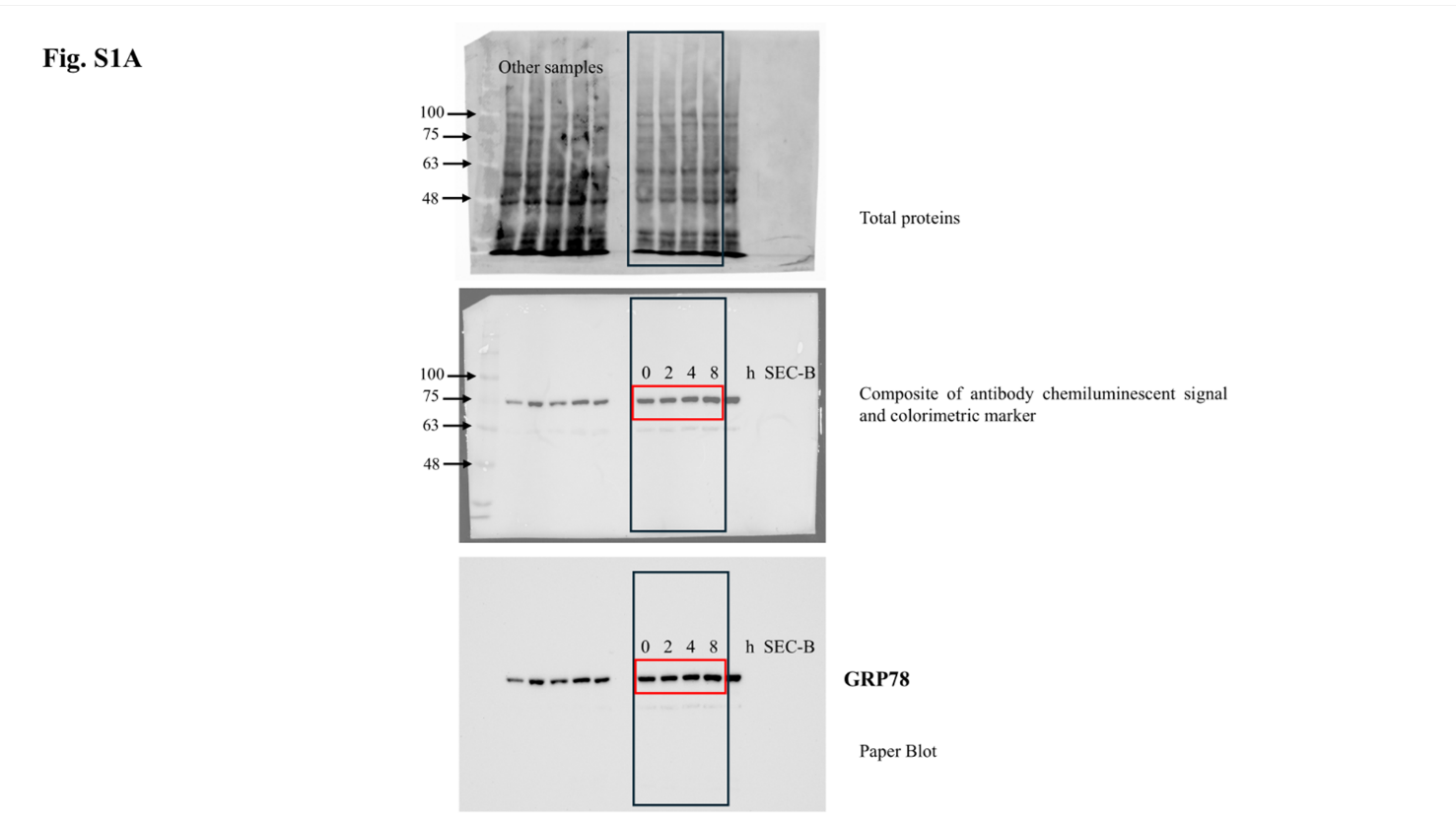

Supplement: Supplementary Materials [file mmc1.docx]
